# The Hidden Burden: Addressing Disparities in Childhood Cancer Incidence Trends

**DOI:** 10.1002/cnr2.70728

**Published:** 2026-11-12

**Authors:** Helal Metwalli, Mohammad Najm Dadam, Lam Ngoc Tram, Tuan Vu Nguyen, Tran Quoc Huy, Tran Cong Duy Long, Nguyen Tien Huy

**Affiliations:** ^1^ Benha University Hospitals Faculty of Human Medicine, Benha University Benha Egypt; ^2^ Online Research Club Nagasaki Japan; ^3^ Department of Orthopedics and Trauma Surgery Marien Hospital Witten Witten Germany; ^4^ Hia Phong University of Medicine and Pharmacy Hai Phong Vietnam; ^5^ Ho Chi Minh City Eye Hospital Ho Chi Minh City Vietnam; ^6^ Huy Quoc Tran College of Health Sciences Nam Can Tho University Can Tho Vietnam; ^7^ Department of Hepatobiliary and Pancreatic Surgery University Medical Center at Ho Chi Minh City Ho Chi Minh City Vietnam; ^8^ Department of Surgery, Faculty of Medicine University of Medicine and Pharmacy at Ho Chi Minh City Ho Chi Minh City Vietnam; ^9^ Institute of Research and Development Duy Tan University Da Nang Vietnam; ^10^ School of Medicine and Pharmacy Duy Tan University Da Nang Vietnam; ^11^ Graduate School of Tropical Medicine and Global Health (TMGH) Nagasaki University Nagasaki Japan

**Keywords:** disparities, incidence, pediatric oncology

## Abstract

**Background:**

Childhood cancer incidence and survival vary substantially worldwide, with major disparities between high‐ and low and middle‐income countries (HICs and LMICs). This review synthesizes global disparities in incidence, survival, and the factors contributing to inequity.

**Recent Findings:**

Childhood cancer incidence has increased in several regions, but trends vary geographically. Major disparities persist in registry coverage, diagnosis, stage at presentation, treatment access, and survival, with 5‐year survival exceeding 80% in most HICs but remaining below 30% in many LMICs.

**Conclusion:**

Global disparities reflect interacting socioeconomic, environmental, genetic, and health‐system factors, compounded by substantial data gaps and inconsistent surveillance. Strengthening cancer registries, standardizing classification and staging, expanding equitable cancer care, and prioritizing multinational research are essential to reduce these inequities.

## 1 Introduction

Pediatric cancer represents a diverse group of malignancies with substantial geographic and temporal variation in incidence. Population‐based cancer registries have become the cornerstone of monitoring these patterns, providing standardized estimates that facilitate comparisons across countries and over time. Recent international registry studies have reported increasing incidence rates in many high‐income countries (HICs), while data from low‐ and middle‐income countries (LMICs) remain limited because of underdiagnosis, incomplete registration and variable diagnostic capacity [1, 2, 3]. Global estimates suggest approximately 400 000 new pediatric cancer cases annually, with leukemia, brain tumors and lymphomas being the most frequently diagnosed. In many HICs, temporal analyses using large datasets such as the SEER program have documented a gradual rise in overall incidence over the past few decades. This observed trend is likely driven by a combination of improved case ascertainment and potential changes in environmental, genetic, or perinatal exposures [4, 5]. Despite improvements in early detection and treatment, survival outcomes vary dramatically. It exceeds 80% in HICs but remains below 30% in many LMICs, which highlights persistent global inequities in cancer care and research capacity [6]. Given these evolving patterns, a comprehensive understanding of global disparities in childhood cancer is essential to guide equitable cancer control strategies.

The objective of this narrative review is to synthesize current evidence on worldwide disparities in childhood cancer, with a particular focus on incidence trends, regional variations, survival outcomes, socioeconomic and health‐system determinants, methodological challenges in disease surveillance and policy implications for improving equity in pediatric oncology.

## 2 Methods

This narrative review synthesized recent evidence from PubMed, Scopus, Web of Science, WHO, GLOBOCAN, SEER, IICC, and other population‐based cancer registries. Studies addressing childhood cancer incidence, survival, disparities, registry quality, and policy implications across HICs and LMICs were prioritized, including systematic reviews, international registry analyses, and major health organization reports.

Findings were narratively synthesized according to epidemiology, regional disparities, determinants of inequity, survival outcomes, methodological challenges, and policy implications. Because this was a narrative review, no formal risk‐of‐bias tool was applied. For the principal incidence studies, we qualitatively considered study design, geographic and population coverage, registry completeness, diagnostic capacity, dependence on statistical modeling, and the likely direction of bias.

### 2.1 Global Epidemiology and Incidence Trends

According to the International Incidence of Childhood Cancer (IICC) project, there was an increase in age‐standardized incidence from approximately 124 per million person‐years in 1981–1990s to 140.6 per million in 2001–2010s. Leukemia, CNS tumors, and lymphomas remained the most common malignancies [1, 2].

Long‐term analyses from the United SEER Program reported a gradual increase in overall childhood cancer incidence over four decades, accompanied by substantial improvements in survival [7]. Similar increases have been observed in Germany through the German Childhood Cancer Registry, particularly for CNS tumors [8], whereas registry studies from Finland reported stable overall incidence with declining rates of malignant CNS tumors during recent decades [9]. In contrast, regional registry data from China demonstrated increasing incidence and mortality, especially in rapidly developing regions [10], while early registry experience from The Gambia identified a markedly different tumor distribution characterized by relatively few CNS tumors and higher proportions of Wilms tumor and leukemia [11]. These incidence estimates originate from national or regional population‐based registries rather than globally representative datasets, which highlights the importance of interpreting observed trends within their respective geographic and methodological contexts. Table 1 summarizes major international studies according to study characteristics, population, principal findings, and key methodological limitations.

**TABLE 1:** Major international studies evaluating childhood cancer incidence and global disparities.

Study	Study design and data source	Registry coverage	Main findings	Risk of bias and methodological limitations	Implications for interpretation
Steliarova‐Foucher et al. (IICC‐3) [1]	International population‐based registry study	153 registries in 62 countries, with uneven LMIC representation	Substantial international variation in incidence. Leukemia and CNS tumors were most common.	Selection of participating registries and unequal completeness and diagnostic capacity across countries.	Strong for standardized international comparison, but reported differences may partly represent registry performance
Johnston et al. [3]	Global model‐based incidence estimation	Global estimates, including countries without adequate registries	Estimated approximately 400 000 childhood cancer cases annually, with substantial underdiagnosis in LMICs.	Dependent on modeling assumptions and extrapolation where observed data were sparse.	Useful for estimating hidden burden, but values should not be treated as directly observed incidence
Wu et al. (GBD) [2]	Global comparative modeling study using GBD data	Global, regional and national estimates	Quantified trends in incidence, mortality and DALYs and persistent HIC and LMIC disparities.	Model specification and source‐data limitations may produce greater uncertainty in data‐poor settings.	Appropriate for broad burden comparisons, with cautious interpretation of country‐level differences
Sultan et al. [7]	Longitudinal population‐based registry analysis	SEER population in the United States over 45 years	Increasing incidence with improved survival.	Changes in ascertainment and classification over time. Limited external generalizability.	Provides relatively strong evidence for a US temporal trend, but not for a global increase
Wellbrock et al. [8]	National population‐based registry analysis	German Childhood Cancer Registry, 1980–2019	Rising CNS tumor incidence over four decades.	Changes in imaging, registration and classification may influence the trend. Country‐specific data.	The increase may combine epidemiological change with improved detection and coding
Abuhamed et al. [9]	National population‐based registry analysis	Finland, 1990–2017	Stable overall incidence with a decline in malignant CNS tumors.	Small annual numbers and changes in tumor behavior coding may affect subgroup trends.	Not directly inconsistent with rising total CNS tumor incidence reported elsewhere
Ji et al. [10]	Regional population‐based registry analysis	Pudong, China, 2002–2015	Increasing childhood cancer incidence over time.	Regional rather than national coverage. Rapid changes in diagnostic access and registration.	The observed increase may partly represent improved ascertainment
Adegoke et al. [11]	Prospective pediatric cancer registry study	The Gambia, first year of registry operation	Relatively few CNS tumors and higher proportions of Wilms tumor and leukemia.	Short observation, small numbers and probable underdiagnosis of tumors requiring imaging or pathology.	Tumor distribution should not be assumed to represent true national incidence

The apparently inconsistent trends should not be interpreted as direct evidence of biological differences between populations. The SEER and German studies used mature registries with long observation periods, although changes in case ascertainment, imaging, and tumor classification may have contributed to the reported increases, particularly for CNS tumors [8, 9]. The stable overall incidence in Finland is not directly inconsistent with the German finding because the Finnish decline was limited to malignant CNS tumors and may be sensitive to changes in behavior coding [10]. The Pudong study covered one rapidly developing region rather than all of China, and improved access to diagnosis and registration may have contributed to its increasing estimates [11]. The Gambian study represented the first year of a prospective registry, and the low proportion of CNS tumors may partly result from limited neuroimaging, pathology, and case ascertainment rather than a true low incidence [12]. Therefore, within‐registry temporal trends from mature population‐based systems are more reliable than direct comparisons of incidence magnitude across settings with different diagnostic and registration capacity.

### 2.2 Major Sources of Childhood Cancer Surveillance Data and Their Implications

National and regional population‐based cancer registries, including the German Childhood Cancer Registry, the Nordic cancer registries, and several emerging registries across Asia, Africa, and Latin America, contribute valuable epidemiological information by monitoring incidence trends, survival outcomes, and the impact of national cancer control programs. However, registry performance varies considerably across countries with respect to population coverage.

The SEER Program is among the world's most comprehensive population‐based cancer registries. Covering approximately half of the United States population, SEER provides high‐quality longitudinal data on cancer incidence, survival, demographics and tumor characteristics using standardized coding systems. Its strengths include rigorous quality control, long‐term follow‐up and widespread use for epidemiological research [12, 13]. However, SEER reflects the US healthcare system and population and therefore may not fully represent disease patterns in countries with different demographic profiles, healthcare access or environmental exposures. The IICC project enables robust international comparisons by applying uniform inclusion criteria and diagnostic classifications. Nevertheless, registry participation remains uneven, with substantially greater representation from HICs than from many LMICs [14].

GLOBOCAN provides global estimates of cancer incidence and mortality for virtually every country. Unlike SEER or IICC, GLOBOCAN combines observed registry data with statistical modeling where national cancer registries are incomplete or unavailable. Consequently, GLOBOCAN offers unparalleled global coverage but its estimates are subject to greater uncertainty in countries lacking high‐quality population‐based registries [15]. In addition to these international initiatives, national and regional population‐based cancer registries, including the German Childhood Cancer Registry, the Nordic cancer registries, and several emerging registries across Asia, Africa, and Latin America, contribute valuable local epidemiological information. These registries often capture region‐specific trends and permit evaluation of national cancer control programs [16, 17]. Table 2 provides a valuable summary of the Major Population‐Based Childhood Cancer Surveillance Systems.

**TABLE 2:** Major population‐based childhood cancer surveillance systems.

Data source	Geographic coverage	Main strengths	Main limitations	Implications for international comparisons
SEER [12, 13]	United States	High‐quality population‐based registry; long follow‐up; standardized coding; detailed survival analyses	Represents only US population	Excellent for trend analyses but limited global generalizability
IICC [1, 14]	International (IARC collaborating registries)	Standardized childhood cancer classification (ICCC); enables cross‐country comparisons	Uneven participation, especially limited LMIC coverage	Best resource for comparing incidence across countries when registry quality is considered
GLOBOCAN [15]	Nearly all countries	Global estimates with extensive geographic coverage	Modeled estimates where registry data are lacking; uncertainty varies	Valuable for global burden estimation but should not be interpreted as directly observed incidence
National Population‐based Registries [16, 17]	Country‐specific	Reflect local epidemiology and health system performance	Variable completeness, coding practices, and diagnostic capacity	Essential for national planning but require harmonization before international comparisons

### 2.3 Regional Differences in Childhood Cancer Burden

Declines in malignant CNS tumors have been reported only in France, Australia, and New Zealand [12]. In Finland, the age‐standardized incidence rate was 4.3 per 100 000 person‐years between 1990 and 2017 [9]. While in Germany, the incidence among children aged 0–14 years increased from 2.86 per 100 000 in the 1990s to 4.3 per 100 000 in the 2010s and become the second most common tumor among German children [8]. Similarly, in China, incidence and mortality from CNS tumors have risen significantly, particularly in rural regions [10]. In contrast, data from the Gambia show CNS tumors are rare (2.3% of pediatric cancers), with Wilms tumor and leukemia being more common [11].

### 2.4 Determinants of Global Disparities

There are multiple factors like socioeconomic, environmental, genetic and health system, which result in these disparities. Socioeconomic status (SES) strongly influences both the likelihood of diagnosis and outcomes. Children from low‐SES families often experience delays in diagnosis, limited inclusion in cancer registries and reduced access to advanced treatment modalities [18]. Environmental and genetic factors also play crucial roles in shaping incidence patterns. Exposure to UV radiation has been implicated in specific pediatric malignancies, particularly melanoma., air pollution, pesticides, and infectious agents like Epstein Barr virus have all been implicated as risk factors for specific pediatric cancers [5, 19, 20, 21]. Additionally, hereditary cancer syndromes including Li‐Fraumeni and Down syndrome account for a small but important subset of cases globally [22, 23]. The cumulative impact of these socioeconomic, environmental and health‐system factors is most evident in survival outcomes, which remain one of the clearest indicators of global inequities in childhood cancer care.

### 2.5 Stage at Diagnosis and Standardized Pediatric Cancer Staging Systems

Stage at diagnosis and standardized staging are complementary but address different dimensions of international disparities. Countries such as Australia, New Zealand, Canada, and participants in the BENCHISTA project have successfully implemented the Toronto Childhood Cancer Stage Guidelines, allowing reliable comparison of stage distributions across registries. These countries generally report high completeness of stage recording (> 90%) and predominantly localized or regional disease at diagnosis for many pediatric solid tumors, reflecting efficient referral pathways and timely diagnostic evaluation [24, 25]. In contrast, studies from Brazil and sub‐Saharan Africa show that although implementation of the Toronto Guidelines is feasible in middle‐ and low‐income settings, children more frequently present with advanced or metastatic disease because of delayed diagnosis, restricted access to specialized oncology services, and limited diagnostic infrastructure. Importantly, standardized staging revealed that poorer outcomes in these regions were associated not only with treatment limitations but also with later disease presentation [26, 27]. The recent BENCHISTA project further demonstrated that even among HICs, differences in stage at diagnosis exist and applying a common staging framework is essential to distinguish genuine epidemiological differences from inconsistencies in registry practices. This represents one of the strongest contemporary examples of how standardized pediatric staging enhances international comparisons.

## 3 Survival Outcomes and Health Inequities

Although incidence trends provide important epidemiological insights, survival remains the most striking indicator of global inequity in childhood cancer. Five‐year survival exceeds 80% in most HICs but remains below 30% in many LMICs because of delayed diagnosis, inadequate diagnostic infrastructure, treatment abandonment, shortages of pediatric oncology specialists, and limited access to essential medicines [28, 29].

### 3.1 Data Gaps and Methodological Challenges and Unresolved Controversies

Despite growing recognition of health disparities in pediatric cancer, significant research gaps and methodological challenges persist, limiting the ability to fully understand and address inequities in childhood cancer care and outcomes.

Primary data gaps include underrepresentation of LMICs and minority populations, disease‐specific and subgroup gaps, and limited focus on reducing disparities. Specifically, in a 2024 scoping review on childhood cancer inequities, Jean Hunleth and colleagues reported that of the 119 studies on childhood cancer identified, 84 were conducted in HICs and only 29 studies examined issues related to race and ethnicity, with the majority involving predominantly or exclusively White children as study participants [30, 31].

A major methodological challenge in pediatric oncology research is the lack of consistent, standardized classification and coding systems, notably the International Classification of Childhood Cancer ICCC‐3 and the International Classification of Diseases for Oncology (ICD‐O). This ICD‐O is a WHO system used for coding the topography and morphology of neoplasms. Its third edition ICD‐O‐3 is the current global standard and forms the basis for the ICCC‐3. These systems ICD‐O‐3 and ICCC‐3 were developed, but their updates and revisions have led to inconsistencies in coding, especially for rare or newly recognized tumor types [1, 32].

The quality of the underlying evidence also differs by data source. International registry studies provide standardized classification and large populations but preferentially include countries with established registries. Model‐based studies extend estimates to countries without adequate registration, but their results depend on assumptions derived partly from better‐documented settings. Long‐standing national registries generally provide the strongest evidence for within‐country trends, although their findings may not be generalizable. Short‐term or regional registries in settings with limited diagnostic services are more vulnerable to missed cases, referral bias, and unstable annual estimates. Consequently, current evidence cannot fully separate true epidemiological variation from differences in diagnosis and registry performance.

Finally, there are some knowledge gaps that can be summarized as follows: first, it is unclear whether the rising global incidence reflects a true biological increase or improvements in diagnosis and cancer registration. Second, the determinants of international incidence variation including genetic, environmental, socioeconomic, and healthcare factors remain poorly understood. Third, standardized data on stage at diagnosis are lacking in many countries despite the availability of the Toronto Childhood Cancer Stage Guidelines. Fourth, LMICs remain underrepresented in population‐based research, resulting in reliance on modeled estimates. Fifth, implementation studies evaluating interventions to reduce disparities are scarce. Sixth, rare pediatric malignancies remain inadequately studied. Seventh, evidence linking environmental exposures to childhood cancer remains inconsistent. Eighth, survivorship outcomes beyond five‐year survival are insufficiently investigated, particularly in LMICs. Ninth, the role of artificial intelligence and digital health in pediatric oncology remains underexplored. Finally, incomplete international harmonization of registry coding, staging and reporting continues to limit the validity and comparability of global epidemiological studies.

### 3.2 Policy and Research Implications

Addressing global disparities in pediatric cancer incidence requires coordinated policy action, sustained investment in data infrastructure, and equitable research partnerships.

Caution is required when interpreting international comparisons because higher reported incidence may reflect better detection rather than a greater underlying disease burden.

First, establishing standardized, interoperable systems aligned with ICD‐O‐3 and ICCC‐3 frameworks, supported by international training and data‐sharing initiatives, would enhance global comparability and surveillance accuracy.

Second, governments should integrate childhood cancer within national cancer control plans, ensuring inclusion in universal health coverage packages. Partnerships with the WHO Global Initiative for Childhood Cancer can help to provide technical support and capacity‐building frameworks.

Third, research priorities must shift from descriptive epidemiology toward solution‐oriented studies that evaluate interventions to reduce inequities.

Future work should examine the impact of socioeconomic, genetic, and environmental determinants through multi‐country, population‐based studies, including underrepresented regions and ethnic groups.

Rather than proposing entirely new policy principles, our review translates the evidence and methodological challenges identified across global and national surveillance systems into a prioritized implementation roadmap. The synthesis indicates that disparities in childhood cancer incidence cannot be addressed through improved treatment access alone; they also require improvements in the quality, completeness, comparability, and geographic representativeness of epidemiological data. Accordingly, the roadmap prioritizes registry strengthening and harmonization in the short term, integration of childhood cancer surveillance and diagnostic capacity into national cancer‐control strategies in the medium term, and sustained multinational research and pediatric oncology infrastructure development in the long term (Table 3).

**TABLE 3:** Roadmap for future plans.

Timeline	Priority	Problem	Expected outcome
Short‐term (1–3 years)	Strengthen cancer registries, adopt ICD‐O‐3/ICCC‐3, improve reporting, train registry staff	Poor‐quality data, inconsistent coding	More accurate incidence estimates and comparable international data
Medium‐term (3–7 years)	Integrate childhood cancer into national cancer control plans and universal health coverage, expand diagnostics and referral networks, strengthen WHO partnerships	Delayed diagnosis, unequal access to care	Earlier diagnosis and improved treatment access
Long‐term (7–15 years)	Invest in pediatric oncology infrastructure, multinational collaborative research, precision medicine, and implementation science	Persistent survival disparities and limited evidence from LMICs	Sustainable reductions in global inequities and improved survival

## 4 Conclusion

Promoting equity‐focused global collaborations, linking cancer registries, policy makers, and pediatric cancer networks, can drive progress toward early diagnosis and equitable care access. By aligning investments in data quality, healthcare infrastructure, and research inclusiveness, the global community can transform the current disparities in childhood cancer incidence into opportunities for shared learning and improved outcomes for all children worldwide.

## Author Contributions

**Helal Metwalli:** writing – review and editing, writing – original draft, conceptualization, validation, visualization, investigation, methodology, project administration. **Mohammad Najm Dadam:** investigation, visualization, writing – original draft, conceptualization, methodology, writing – review and editing. **Lam Ngoc Tram:** writing – review and editing, writing – original draft. **Tuan Vu Nguyen:** project administration, resources, writing – review and editing. **Tran Quoc Huy:** writing – original draft, writing – review and editing. **Tran Cong Duy Long:** supervision, writing – original draft, conceptualization, validation, project administration, writing – review and editing, methodology, investigation. **Nguyen Tien Huy:** investigation, writing – review and editing, project administration, supervision, data curation, validation, visualization, methodology, writing – original draft, conceptualization.

## Funding

The authors have nothing to report.

## Conflicts of Interest

The authors declare no conflicts of interest.

## Data Availability

All data analyzed in this study were obtained from previously published literature and publicly available sources. The data sources are appropriately cited in the manuscript. No new datasets were generated or collected specifically for this study.
